# Long-term outcomes of patients with breast cancer after nipple-sparing mastectomy/skin-sparing mastectomy followed by immediate transverse rectus abdominis musculocutaneous flap reconstruction: Comparison with conventional mastectomy in a single center study

**DOI:** 10.1097/MD.0000000000010680

**Published:** 2018-05-04

**Authors:** Sae Byul Lee, Jong Won Lee, Hee Jeong Kim, Beom Seok Ko, Byung Ho Son, Jin Sup Eom, Taik Jong Lee, Sei-Hyun Ahn

**Affiliations:** a Division of Breast and Endocrine Surgery, Department of Surgery; b Department of Plastic Surgery, Asan Medical Center, University of Ulsan College of Medicine, Seoul; c Department of Plastic Surgery, Boryeong Asan Medical Center, Boryeong, Korea.

**Keywords:** breast cancer, local recurrence, mastectomy, reconstruction, survival

## Abstract

To evaluate the oncological outcomes of patients with breast cancer after nipple-sparing mastectomy (NSM)/skin-sparing mastectomy (SSM), followed by immediate reconstruction, as compared to conventional mastectomy (CM).

SSM/NSM has been increasingly used to treat women with breast cancer who wish to preserve the overlying breast skin, but concern exist regarding its oncological safety due to the potential for residual breast tissue. We report our experience performing SSM/NSM for breast cancer treatment compared to CM with a long follow-up period.

All consecutive patients who underwent mastectomy for breast cancer at Asan Medical Center between January 1993 and December 2008 were identified by retrospective medical chart review. The patients who underwent NSM/SSM, followed by immediate breast reconstruction with a pedicled transverse rectus abdominis musculocutaneous flap (TRAM), were compared to the patients who underwent CM in terms of breast-cancer specific survival (BCSS) rate, distant metastasis-free survival (DMFS) rate, and local recurrence (LR) rate.

During the study period, 6028 patients underwent mastectomy for breast cancer. Of these, 1032 and 4996 underwent NSM/SSM with TRAM and CM, respectively. Their median follow-up durations were 94.4 (range, 8.1–220.2) and 110.8 (range, 6.1–262.0) months, respectively. Their 5 year BCSS rates were 95.4% and 88.1%, respectively (log-rank, *P < *.001). Their 5 year DMFS rates were 93.0% and 85.6%, respectively (log-rank, *P < *.001).

Relative to CM, NSM/SSM, followed by immediate breast reconstruction, may be a viable and oncologically safe surgical treatment in selected patients with breast cancer.

## Abstract 2

Supplemental Digital Content is available in the text

## 1 Introduction

Breast cancer is the second most common malignancy among women, and its incidence in Korea is increasing rapidly.^[1]^ This increase reflects to some degree the advent of breast-cancer screening strategies. As a result, many of the breast cancers that are currently detected are in the early stages.^[2,3]^ However, at the time of diagnosis, many patients still present with diffuse microcalcification or multifocal or multicentric breast cancer. Some also have advanced-stage breast cancer. Although breast-conserving surgery is increasingly being used to treat breast cancer cases, about one-third of women with breast cancer still must undergo mastectomy due to the size of the tumor, the site of the lesion, and/or the extent of the tumor.^[4–9]^ Recently in cases of breast cancers, mastectomy with complete preservation of the skin envelope or nipple has been developed to improve aesthetic and psychological outcomes for patients.^[10]^

Skin-sparing mastectomy (SSM) with immediate breast reconstruction (IBR) was first reported by Freeman in 1962 and was modified by Toth and Lappert.^[11]^ It is generally acknowledged to be the method that can achieve both a radical cure and resolve cosmetic issues and has become a common and widely used procedure for patients with ductal carcinoma in situ and early-stage breast cancer.^[12]^ Nipple-sparing mastectomy (NSM) with immediate reconstruction is also widely used for cases where there are no tumors beneath the subareolar area. Several recent series show that NSM and SSM with IBR are oncologically safe.^[13,14]^ However, because less skin is resected in SSM compared to conventional mastectomy (CM), concerns about its oncological safety persist: it is feared that SSM could increase the risk of local, regional, or systemic breast cancer recurrence.^[15,16]^ Moreover, the possibility of using SSM for advanced-stage breast cancer has not been thoroughly investigated.^[17,18]^ In addition, most of the studies that investigated SSM with immediate reconstruction suffer from limitations: some involved patient selection (either early stage patients or advanced-stage patients were included), others were small case series, while others utilized short follow-up periods, lacked control groups, or employed more than one type of reconstruction method.^[12,19]^

Previously, our center reported on the oncological safety of SSM/NSM, followed by IBR.^[20]^ That study involved 520 patients with a median follow-up duration of 60 months. The local recurrence (LR) rate was 1.2%. The present study is an extension of that oncological safety study: it compared NSM/SSM, followed by immediate transverse rectus abdominis musculocutaneous (TRAM) flap reconstruction, to CM in terms of local and systemic recurrence and survival rates in a much larger cohort with a long follow-up period.

## 2 Patients and methods

### 2.1 Subjects

All consecutive patients with breast cancer who underwent surgery at Asan Medical Center between January 1993 and December 2008 were identified by a retrospective medical chart review. The patients who underwent mastectomy for breast cancer were selected. Of these, patients were excluded if they received neoadjuvant systemic therapy, had distant metastasis at the time of diagnosis, had a short follow-up period (<6 months), or only underwent breast-conserving surgery and axillary operation. Although all patients with clinical stage 0–III breast cancer had been offered the option of NSM or SSM, followed by immediate reconstruction, as an alternative to CM, only the patients who underwent immediate TRAM flap reconstruction were included in this study. The indications for SSM or NSM were any stage, any tumor size, and any tumor areola distance with indications for mastectomy. Patients with a clinically normal nipple and no skin involvement were offered the option of NSM.

The following data were extracted from the medical records and pathology reports: age at diagnosis, type of surgery, type of adjuvant systemic treatment, histological grade, nuclear grade, the presence of lymphovascular invasion (LVI), and histological subtype, namely, positivity for estrogen receptor (ER), progesterone receptor (PR), or tissue human epidermal growth factor receptor-2 (HER-2). ER status, PR status, and HER-2 status were determined immunohistochemically (IHC) (supplementary data). ER and PR were considered to be positive, if >10% of cells showed positivity. For HER-2 overexpression analysis, cases graded 0, 1+ were considered as negative. And cases graded 2+ were not evaluated by fluorescence in situ hybridization, and regarded negative, 3+ result for that was considered positive. Tumor staging was performed according to the tumor-node-metastasis classification of the 7th American Joint Committee on Cancer. This study was reviewed and approved by the Institutional Review Board of Asan Medical Center (20150049). Due to the retrospective nature of the study, the requirement for informed consent was waived.

After surgery, the patients were regularly followed-up every 3 to 6 months for the first 5 years and every 12 months thereafter. Relapse and metastasis were identified on the basis of clinical examination, mammography, chest radiography, and tumor marker (CA15–3) measurements, which were performed at every follow-up visit. Abnormal clinical findings might be evaluated by further studies, including chest computed tomography, bone scan, and liver ultrasonography. Patients who failed to present for examination were called on the telephone to confirm that they were still alive.

### 2.2 Statistical analysis

Breast-cancer specific survival (BCSS) was defined as the time from surgery to the time to death due to breast cancer. Distant metastasis-free survival (DMFS) was defined as the time from surgery to the first appearance of distant metastasis. The different surgical method groups were compared in terms of categorical variables by using the Chi-squared test and in terms of continuous variables such as age by using the unpaired Student's *t* test. And, ANOVA test was used in comparing 3 groups for statistical significance. Survival curves were generated by using the Kaplan–Meier method. The significance of differences in survival was tested by using the log-rank test. The Cox proportional-hazards model was used to evaluate the independent prognostic effect of surgical method on BCSS and DMFS with adjustment for other routinely used prognostic factors, namely, age, tumor size, lymph node status, grade, LVI, ER/PR status, tissue HER-2 status, adjuvant chemotherapy, adjuvant radiotherapy, and adjuvant antihormonal treatment. All statistical analyses were performed by using SPSS version 18.0 (SPSS, Chicago, IL). *P < *.05 was considered to indicate statistical significance.

## 3 Results

### 3.1 Patient characteristics

In total, 11,085 patients with breast cancer underwent surgery at Asan Medical Center between January 1993 and December 2008. Of these, 6028 underwent mastectomy: 4996 underwent CM and 1198 underwent NSM/SSM, followed by immediate reconstruction. Of the latter patients, 1032 underwent NSM/SSM, followed specifically by TRAM flap reconstruction. These patients and the whole CM group (n = 4996) were enrolled in this study (Supplementary Fig. 1).

The mean age of the whole cohort was 48.1 ± 10.2 years (range, 23–90 years). Of these 6028 patients, 446 (7.4%), 1550 (25.7%), 2924 (48.5%), and 1108 (18.4%) had stage 0, I, II, and III cancer, respectively. Lymph node metastasis was detected in 2780 patients (46.1%) and 3613 (64.4%) patients were hormone receptor positive. Tissue HER-2 positivity was detected by IHC in 1586 patients (34.4%). LVI was detected in 1089 patients (32.9%) and 1736 (42.0%) were nuclear grade 3 (Table 1).

**Table 1:**
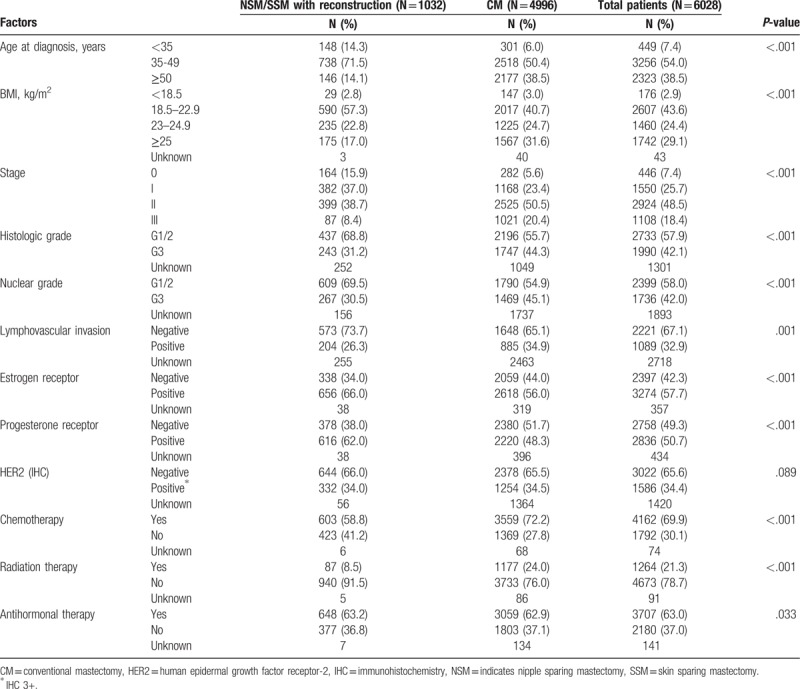
Clinicopathologic characteristics of enrolled patients.

Of the 6028 patients, 1032 (17.1%) underwent NSM/SSM with immediate TRAM flap reconstruction (338 underwent NSM and 694 underwent SSM) and 4996 patients (82.9%) underwent CM. The NSM/SSM group had a statistically significant smaller BMI than the CM group (*P < *.001). The NSM/SSM patients were also significantly younger (*P < *.001) and had an earlier stage (*P < *.001), a lower histological grade (*P < *.001), a lower nuclear grade (*P < *.001), and less LVI (*P* = .001) than the CM group. The NSM/SSM patients were also more likely to be ER- and PR-positive (*P < *.001), less likely to undergo chemotherapy and radiation therapy (*P < *.001), and more likely to receive antihormonal therapy (*P = *.033) (Table 1).

### 3.2 Recurrence patterns

For the NSM/SSM and CM groups, the median follow-up durations were 94.4 (range, 8.1–220.2) and 110.8 (range, 6.1–262.0) months, respectively, and the median times to first recurrence were 37.3 (range, 5.7–157.7) and 32.3 (range, 0.2–255.0) months, respectively. The overall recurrence rates were 15.3% (158/1032) and 24.3% (1214/4996), respectively: this difference was statistically significant (*P < *.001) (Table 2). While the NSM/SSM group had a significantly higher LR rate (3.4%; 35/1032) than the CM group (2.0%; 102/4996) (*P* = .008), the NSM/SSM group also had a lower systemic recurrence rate (8.4%; 87/1032) than the CM group (18.6%; 929/4996) (*P < *.001). The 2 groups did not differ significantly in terms of regional recurrence rate (*P = *.785).

**Table 2:**
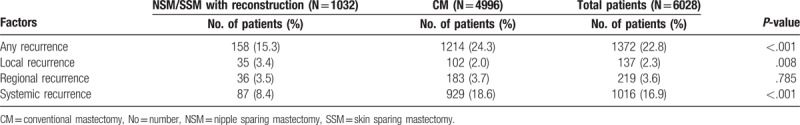
Recurrence patterns according to type of surgery.

**Figure 1:**
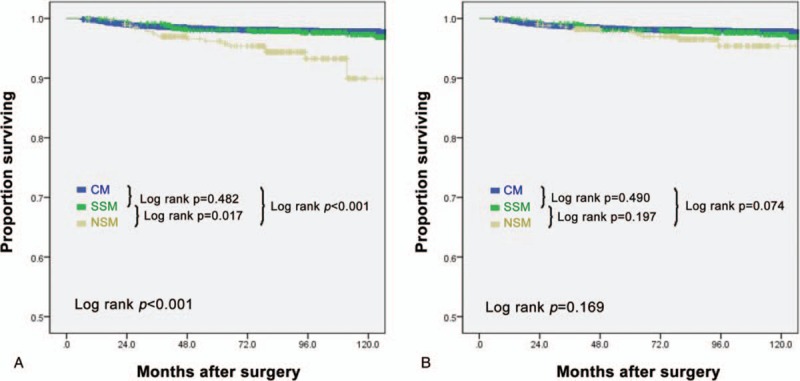
Local recurrence free survival curves for NSM and SSM with reconstruction and CM in 6028 patients. (A**)** Total patients (B**)** Except of nipple areola complex recurrence. (blue line, CM; green line, SSM with reconstruction; yellow line, NSM with reconstruction). CM = conventional mastectomy, NSM = indicates nipple sparing mastectomy, SSM = skin sparing mastectomy.

To determine the cause of the high LR rate in the NSM/SSM group, the LRs associated with each type of surgery were examined (Table 3). The NSM group had a higher LR rate (5.4%; 18/338) than the CM (2.0%; 102/4996) and SSM (2.4%; 17/694) groups (*P < *.001 by Chi-squared test). The SSM and CM groups did not differ significantly in terms of this variable (*P* = .478, data not shown). Also, when all the 3 groups were compared by Kaplan–Meier, the CM, SSM, and NSM groups had 5 year local recurrence free survival rates of 98.2%, 98.0%, and 96.2%, respectively (log-rank, *P < *.001), and these differences were due to a high LR rate in the NSM group (CM vs NSM, *P < *.001; SSM vs NSM, *P* = .017; CM vs SSM, *P = *.482) (Fig. 1A). NSM differs from SSM and CM in that the nipple-areola complex (NAC) is preserved, and the NSM group was found to have 7 cases of recurrence in the NAC. To assess whether this contributed particularly to the difference between the NSM, SSM, and CM groups in terms of LR rates, we excluded the NAC recurrences in the NSM group from the analysis. Indeed, the 3 groups no longer differed significantly in terms of overall LR rates (*P = *.284 by ANOVA). The NSM, SSM, and CM groups were also compared to determine whether their LRs associated with specific cancer stages or subtype. However, such associations were not detected (Supplementary Table 1). Also, when an analysis of local recurrence free survival by Kaplan–Meier, 5 year local recurrence free survival rates were significantly not different for the all 3 groups (log-rank, *P = *.169) (Fig. 1B). The characteristics of the 7 patients with NAC recurrence are shown in Table 4. After the NAC recurrence, all underwent NAC excision. Additional locoregional or systemic recurrence was not detected during the follow-up periods of these patients.

**Table 3:**
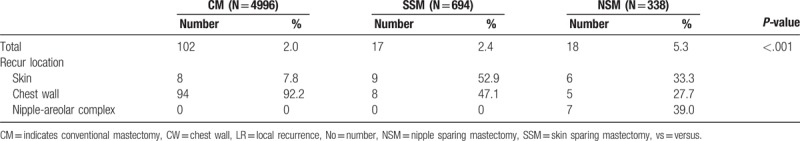
Local recurrences according to type of surgery (CM vs SSM vs NSM).

**Table 4:**
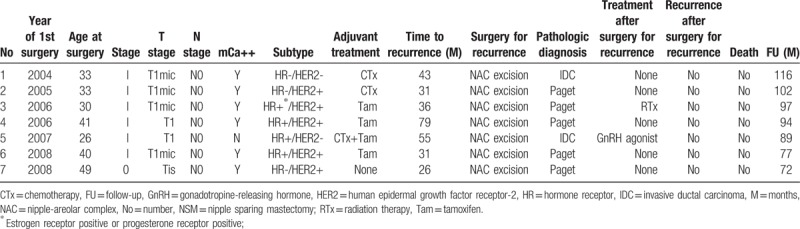
Description of 7 patients underwent NSM followed by immediate reconstruction with NAC recurrence.

### 3.3 Survival analysis

**Figure 2:**
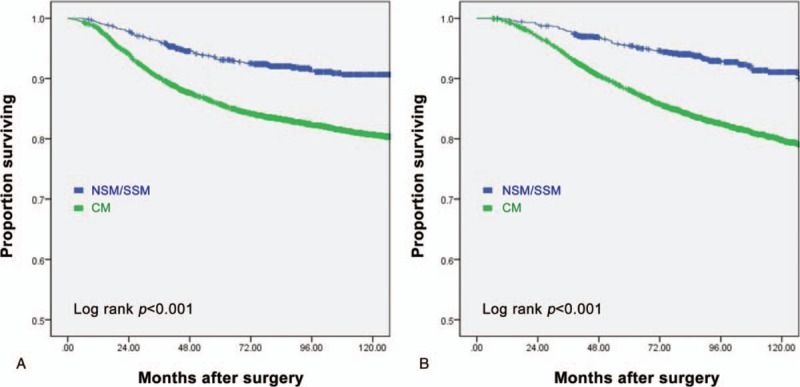
Survival curves for NSM/SSM with reconstruction and CM in 6028 patients. (A) Distant metastasis-free survival (DMFS), (B) Breast cancer-specific survival (CSS). Green line, NSM/SSM; blue line, CM. CSS = cancer-specific survival, CM = conventional mastectomy, NSM = nipple sparing mastectomy, SSM = skin sparing mastectomy.

The NSM/SSM and CM groups had 5 year DMFS rates of 93.0% and 85.6%, respectively (log-rank, *P < *.001), and 5 year BCSS rates of 95.4% and 88.1%, respectively (log-rank, *P < *.001) (Fig. 2). Univariate analysis showed that NSM/SSM associated significantly with a higher DMFS and BCSS than CM. However, multivariate analysis revealed that surgery method did not associate significantly with DMFS or BCSS. Instead, a younger age, larger tumor size (>2 cm), higher nuclear grade, presence of LVI, ER negativity, and positive lymph nodes were found to associate with a worse DMFS and BCSS (Table 5). To overcome selection bias, we performed 1:1 propensity score (PS) matching between the CM and NSM/SSM cohorts. After PS matching, 896 patients were included in each group. The CM and NSM/SSM groups were well matched for age at diagnosis, period at operation, BMI, stage, histologic grade, nuclear grade, ER, PR, LVI, HER-2 status, and adjuvant treatment including chemotherapy, hormonal therapy, and radiotherapy. Despite PS matching, univariate analysis showed that NSM/SSM associated significantly with a higher DMFS and BCSS than CM (DMFS: HR = 0.74, 95% CI = 0.547–0.995, *P = *.047; BCSS: HR = 0.74, 95% CI = 0.544–0.995, *P = *.046; supplementary data).

**Table 5:**
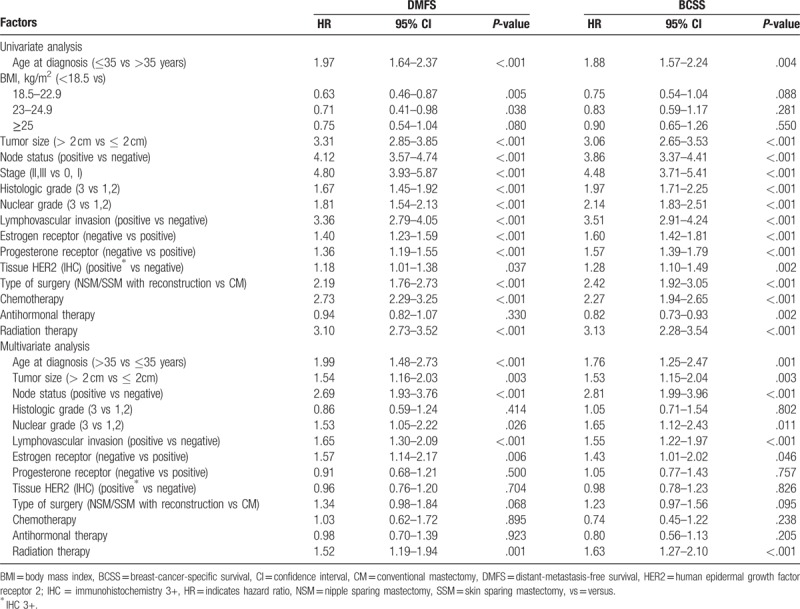
Cox proportional hazard model analysis for distant-metastasis-free survival (DMFS) and breast-cancer-specific survival (BCSS).

## 4 Discussion

In oncoplastic surgery, both the oncological and cosmetic outcomes are important objectives. Cosmetic outcomes are increasingly being considered because the chance of dying from breast cancer has fallen. This is due to advances in screening programs, our greater understanding of cancer biology, and the development of new treatments. These advances mean that the quality of life after surgery can now be included in the treatment decision-making process.^[3,5]^

Over the last decade, SSM for patients with early stage breast cancer has become a common and widely used procedure.^[12]^ It involves removing the entire breast while preserving the skin envelope and the natural infra-mammary fold; when combined with IBR, it yields an improved cosmetic result.^[21]^ Moreover, because the anesthetic risk is lower these days, and the emotional trauma felt by the patient because of the loss of their breast is reduced, SSM followed by IBR is ultimately cost effective.^[22]^ However, the fact that most or all of the native breast skin is left intact during SSM led some authors to speculate that SSM may be oncologically inferior to CM as it may yield a high LR rate.^[23]^ Prospective data comparing the rates of local and regional recurrence after SSM and CM are not available. However, several retrospective series demonstrated the oncological safety of SSM with IBR. The SSM-associated LR rates reported by these studies are listed in supplementary Table 2.^[14,21,23–38]^ These reviews with median follow-up times ranging from 26 to 117 months reported similar LR rates for patients undergoing SSM (0%–6.2%) and CM (0.8%–4.0%). In addition, a meta-analysis of 7 observational studies that compared SSM to CM in breast cancer reported that SSM did not differ significantly from CM in terms of LR rates.^[28]^ However, the reported LR rates vary widely, and the adjuvant treatment data in these studies are incomplete or absent. Moreover, these studies suffer from some limitations, as follows.

First, many of the initial studies of SSM only included patients with in situ or early stage invasive disease.^[24,29,30,39]^ These studies also only included patients who were treated incipient periods, which is when most surgeons still had relatively little experience with SSM. Several other studies that limited the study population to SSM-treated patients with locally advanced breast cancer reported LR rates of 1% and 10%.^[18,19,40]^ In our study, 39.3% of the SSM-treated patients had IIB or higher stages and we observed a comparable LR rate for stage IIB and III patients (8/241, 3.3%). Moreover, the patients with locally advanced disease who underwent SSM and CM had similar LR rates (Supplementary Table 1). The fact that our series involved a relatively large proportion of patients with stage IIB and III disease as well as early stage disease and yet found no significant difference from CM in terms of LR rates supports the rational use of SSM with immediate TRAM flap reconstruction in patients.

The second limitation of the previous studies on SSM versus CM was that they generally involved short follow-up periods. This did not initially seem to be a major limitation because the study by Crowe and colleagues on 1392 breast cancer patients who underwent mastectomy showed that locoregional recurrence occurred within the first 3 years in most cases, with a peak being observed in the second year.^[41]^ The recurrence rate remained relatively constant over a long period with a sharp decrease. However, local recurrence has been reported 10 years after mastectomy, which means that follow-up is needed for > 10 years.^[14,42]^ This is supported by several studies from the MD Anderson Cancer Center. First, an early report in 1996 reported an overall regional recurrence (RR) rate of 2.6% in 545 patients who underwent SSM and IBR.^[43]^ However, analysis of the subset of 95 patients who were followed-up for more than 4 years revealed that their RR rate was substantially higher at 4.2%. Similarly, the 104 patients from the same cohort who were followed-up for more than 5 years had a LR rate of 6.7%,^[44]^ but a second report from the same center showed that when only patients with at least 6 years of follow-up were included,^[24]^ the LR rate of the 114 SSM patients were somewhat higher at 7.0%. These series of publications from the same center adeptly demonstrate the importance of long-term follow-up in determining the true recurrence rates after breast cancer surgery. In our study, the median follow-up duration of the whole cohort was 106.8 (range, 6.1–262.0) months. Since the median time to LR was 49.9 (range, 0.9–247.6) months, LR rate that occurs 5 years after mastectomy was 39.4% (54/137).

In our study, the LR rate of the SSM/NSM group was 3.4% (35/1032), which is similar to previous reports. However, the CM group had a significantly lower LR rate (2.0%, 102/4996) (*P = *.011) (Table 2). ANOVA of LR rates of the SSM, NSM, and CM groups also detected a significant difference (*P < *.001) (Table 3). However, univariate analyses revealed that these differences were due to a high LR rate in the NSM group (CM vs NSM, *P < *.001; SSM vs NSM, *P = *.017; CM vs SSM, *P = *.482) (Fig. 1A). The closer examination of the NSM group recurrences revealed that of the local recurrences in this group, seven (2.1%, 7/338) involved the NAC. When these NAC cases were excluded from the analysis, the LR rates of the SSM/NSM and CM groups were 2.7% and 2.0%, respectively, and no longer differed significantly (*P = *.284) (Supplementary Table 1). Moreover, when all the 3 groups (i.e., SSM, NSM, and CM) were compared by Kaplan–Meier, the previous statistically significant difference was lost (*P = *.169) (Fig. 1B).

Several studies showed that generally, about 3% to 10% of breast cancer cases have tumor cells in the NAC; the one exception was the study that reported the extremely high percentage of 58%.^[4,22,45,46]^ Factors that dictate NAC involvement include the size of the primary breast tumor, its distance from the NAC, multicentricity, lymph node positivity, and the presence of an extensive intraductal component.^[20]^ In the present study, the indications for NSM were any stage, any tumor size, and any tumor areola distance. The NAC was preserved when palpation and the appearance of the nipple were normal and the intraoperative frozen biopsy result showed no tumor at the subareolar resection margin. For the NSM group in the present study, the rate of recurrence at the NAC was 2.1% (7/338), which is similar to the results of earlier studies. The mean time to NAC recurrence in the 7 patients was 43 (range, 26–79) months after surgery. In the patients with NAC recurrence, the pathological stage was 0 (one patient) and I (six patients), and the 5 patients had Paget's disease. These patients were treated by using wide excision and were free of disease at their last follow-up (Table 4). It is now widely known that not only the LR rate but also the overall survival in SSM is comparable to those in CM, at least for stages 0, I, and II. This is supported by our study: the NSM/SSM group had a higher 5 year BCSS rate (95.4%) than the CM group (88.1%; *P < *.001). The SSM/NSM group also had a higher DMFS rate (93.0% vs 85.6%; *P < *.001). This may be due to selection bias that allows the NSM/SSM with reconstruction group had better prognostic factors. However, after validation with other routinely used prognostic factors in multivariate analysis, type of surgery no longer associated significantly with DMFS and BCSS (Table 5). However, the factors that did associate significantly with DMFS and BCSS on multivariate analysis were a young age, large tumor size (>2 cm), high nuclear grade, the presence of LVI, ER negativity, and positive lymph nodes.

In our study, the patients in the SSM/NSM group were younger on average (42 years) than those in the CM group (49 years). Other studies also found that patients undergoing SSM/NSM tend to be younger, with a mean age of 41 years, compared to which mean age for CM.^[23,30]^ This indicates that these studies suffer particularly from selection bias, namely, the tendency to prefer SSM/NSM for less extensive or lower grade tumors and younger patients. This could reflect a selection bias on the part of surgeons, who may be more likely to offer SSM/NSM to younger patients than to older patients. However, it is more likely that it reflects the disease stage: CM may be favored for high-stage disease rather than a method that involves reconstruction because of the anticipated likelihood that adjuvant radiotherapy will be needed, which is known to compromise reconstruction. Indeed, in the present study, the SSM/NSM with IBR group received less radiotherapy than the CM group (*P < *.001).

The main limitation of the present study was that it involved retrospective analysis. However, its strengths include not only the large number of cases but also the long follow-up time, which was much longer than that employed by previous studies. Other strengths were that the study involved a single institute, only one type of reconstruction method (TRAM flap), and a standardized management protocol.

In conclusion, the present study supported the notion that SSM/NSM does not pose a higher risk of local, regional, or systemic recurrence relative to CM. In particular, our study showed that SSM, followed by IBR using TRAM flap, was an oncologically safe procedure. Thus, SSM may be an attractive alternative to CM. Moreover, NSM may be a viable surgical option for breast cancer patients who lack tumor cells in the NAC. The authors thank all clinical surgeons for collecting data.

## Acknowledgments

The authors thank all clinical surgeons for collecting data. SBL and SHA substantially contributed to the conception and design of the study, and to the analysis and interpretation of the data; SBL drafted the paper, and all authors revised the paper for intellectual content and approved the final version of the paper.

## Author contributions

**Conceptualization:** Sei-Hyun Ahn.

**Data curation:** Jin Sup Eom, Taik Jong Lee.

**Supervision:** Byung Ho Son, Jong Won Lee, Beom Seok Ko, Hee Jeong kim.

**Writing – original draft:** Sae Byul Lee.

## Supplementary Material

Supplemental Digital Content
