# Supplementary material for: Long-term outcomes of patients with breast cancer after nipple-sparing mastectomy/skin-sparing mastectomy followed by immediate transverse rectus abdominis musculocutaneous flap reconstruction: Comparison with conventional mastectomy in a single center study
Source: Medicine (Baltimore). 2018 May 4;97(18):e0680. doi: 10.1097/MD.0000000000010680 (PMC6393080; doi:10.1097/MD.0000000000010680)
Supplement: Supplemental Digital Content [file medi-97-e0680-s001.docx]

Supplementary Figure 1. Consort diagram of enrolled patients. (Total 6,028 patients)

Supplementary Figure 2. Immunohistochemistry of estrogen receptor (ER) and human epidermal growth factor receptor-2 (HER-2). **A** ER negative, **B** ER positive, **C** HER-2 negative, **D** HER-2 positive

Supplementary Figure 3. Survival curves for NSM/SSM with reconstruction and CM in propensity score matched patients. **A** Breast cancer-specific survival(CSS), **B** Distant metastasis-free survival(DMFS). Red line, NSM/SSM; Black line, CM

SUPPLEMENTARY TABLE 1. Local Recurrences According to Type of Surgery (CM vs. SSM vs. NSM except NAC recurrence)

|  | CM | |  | SSM | |  | NSM | | *p-value* |
| --- | --- | --- | --- | --- | --- | --- | --- | --- | --- |
|  | Number of patients | No of LR (%) |  | Number of patients | No of LR (%) |  | Number of patients | No of LR (%) |  |
| Total | 4,996 | 102(2.0) |  | 694 | 17(2.4) |  | 338 | 11(3.3) | 0.284 |
| Stage |  |  |  |  |  |  |  |  |  |
| 0 | 282 | 2(0.7) |  | 110 | 4(3.6) |  | 54 | 0(0.0) | 0.110 |
| I | 1,168 | 17(1.5) |  | 250 | 4(1.6) |  | 132 | 4(3.0) | 0.396 |
| II | 2,525 | 56(2.2) |  | 272 | 7(2.6) |  | 127 | 7(5.5) | 0.059 |
| III | 1,021 | 27(2.6) |  | 62 | 2(3.2) |  | 25 | 0(0.0) | 0.682 |
| Subtype |  |  |  |  |  |  |  |  |  |
| HR+^*^/HER2- | 1,707 | 27(1.6) |  | 351 | 8(2.3) |  | 184 | 7(3.8) | 0.089 |
| HR+/HER2+ | 567 | 16(2.8) |  | 114 | 4(3.5) |  | 60 | 1(1.7) | 0.784 |
| HR-/HER2+ | 683 | 8(1.2) |  | 107 | 1(0.9) |  | 51 | 2(3.9) | 0.233 |
| HR-/HER2- | 671 | 11(1.6) |  | 71 | 3(4.2) |  | 38 | 1(2.6) | 0.304 |
| CM indicates conventional mastectomy; SSM, skin sparing mastectomy; NSM, nipple sparing mastectomy; vs, versus; No, number; LR, local recurrence; CW, chest wall; NAC, Nipple-areolar complex, HR, hormone receptor;  ^*^ estrogen receptor positive or progesterone receptor positive; HER2, human epidermal growth factor receptor-2 | | | | | | | | | |

SUPPLEMENTARY TABLE 2. Local Recurrence Rates After Skin Sparing Mastectomy with Immediate Reconstruction in Previously Published Studies

| Authors et al. | SSM vs. CM (n): FU (follow-up) in months (m) | LR rate (%) |
| --- | --- | --- |
| Newman et al., 1998 | 372 SSM; median FU = 26m | 6.2% |
| Toth et al., 1999 | 50 SSM; median FU = 51.5m | 0% |
| Medina-Franco et al., 2002 | 173 SSM; median FU = 73m | 4.5% |
| Carlson et al., 2003 | 539 SSM; median FU = 61.6m | 5.5%(0.6, 3.0, 10.4, 11.1, 0% in Stage 0, I, II, III, IV respectively) |
| Drucker-Zertuche et al., 2007 | 105 SSM; median FU = 51m | 1% |
| Vaughan et al., 2007 | 210, 206 SSM; median FU = 58.6m | 5.3%(9 of 11 in the index quadrant) |
| Lanitis et al., 2010  Meta-analysis of 7 studies | 825 SSM vs. 2518 CM; median FU for studies = 37.5-101m | 5.7% SSM (3.8-10.4) vs. 4.0% CM(1.7-11.5) |
| Kinoshita et al., 2011 | 73 SSM vs. 129 CM; mean FU = 30m | 2.7% SSM vs. 3.9% CM |
| Nava et al., 2011 | 77 SSM; median FU = 36m | 0.5%/year |
| Sheikh et al., 2011 | 177 SSM; median FU = 28m | 1.1% SSM vs. 0.8% CM (non-significant) |
| Peled et al., 2012 | 126 SSM; median FU = 28m | 2.4% |
| Romics et al., 2012 | 207 SSM; median FU =119(14-163)m | 2.9% |
| Missana et al., 2013 | 400 SSM; median FU = 88 m | 3.0% |
| Van Mierlo et al., 2013 | 157 SSM; median FU = 39 m | 2.9% |
| Liang et al., 2013 | 249 SSM; median FU = 53m | 3.2% |
| **Lee et al., 2015** | **1,032 SSM/NSM; median FU=94(8-220)m** | **3.4% (2.4% SSM vs. 5.3% NSM vs. 2.0% CM)** |
| SSM indicates skin sparing mastectomy; NSM, nipple sparing mastectomy, CM, conventional mastectomy; FU, follow-up; m, months; LR, local recurrence. | | |

SUPPLEMENTARY TABLE 3.ClinicopathologicCharacteristics of PS matched Patients

| Factors |  | NSM/SSM with reconstruction (N=896) | CM (N=896) | p-value |  |
| --- | --- | --- | --- | --- | --- |
|  |  | N (%) | N (%) |  |  |
| Age at diagnosis (years) | Mean (S.D) | 43.23(7.38) | 43.12(6.88) | 0.763 |  |
| BMI (kg/m^2^) | Mean (S.D) | 22.65(3.71) | 22.70(2.97) | 0.768 |  |
| Stage | 0  I  II  III | 99(11.0)  327(36.5)  396(44.2)  74(8.3) | 120(13.4)  317(35.4)  373(41.6)  86(9.6) | 0.289 |  |
| Histologic grade | G1/2  G3  Unknown | 481(66.6)  242(33.4)  173 | 471(66.9)  233(33.1)  192 | 0.772 |  |
| Nuclear grade | G1/2  G3  Unknown | 495(65.2)  264(34.8)  137 | 500(66.4)  250(33.4)  146 | 0.949 |  |
| Lymphovascular invasion | Negative  Positive  Unknown | 501(73.1)  184(26.9)  211 | 483(72.2)  186(27.8)  227 | 0.630 |  |
| Estrogen receptor | Negative  Positive  Unknown | 299(34.8)  561(65.2)  36 | 300(34.8)  562(65.2)  34 | 0.912 |  |
| Progesterone receptor | Negative  Positive  Unknown | 358(41.7)  501(58.3)  37 | 346(40.0)  516(60.0)  34 | 0.772 |  |
| HER2(IHC) | Negative  Positive^*^  Unknown | 556(66.4)  281(33.6)  59 | 552(65.4)  292(34.6)  52 | 0.716 |  |
| Chemotherapy | Yes  No  Unknown | 567(63.6)  324(36.4)  5 | 554(62.2)  336(37.8)  6 | 0.795 |  |
| Radiation therapy | Yes  No  Unknown | 71(8.0)  820(92.0)  5 | 87(9.8)  804(90.2)  5 | 0.411 |  |
| Antihormonal therapy | Yes  No  Unknown | 591(66.3)  300(33.7)  5 | 575(64.8)  314(35.2)  7 | 0.647 |  |
| NSM indicates nipple sparing mastectomy; SSM, skin sparing mastectomy; CM, conventional mastectomy; HER2, Human Epidermal growth factor Receptor-2; IHC, immunohistochemistry  ^*^ IHC 3+ | | | | | |
